# A high-throughput method to detect RNA profiling by integration of RT-MLPA with next generation sequencing technology

**DOI:** 10.18632/oncotarget.17551

**Published:** 2017-05-02

**Authors:** Jing Wang, Xue Yang, Haofeng Chen, Xuewei Wang, Xiangyu Wang, Yi Fang, Zhenyu Jia, Jidong Gao

**Affiliations:** ^1^ Department of Breast Surgical Oncology, National Cancer Center/Cancer Hospital, Chinese Academy of Medical Sciences and Peking Union Medical College, Beijing, China; ^2^ Institute of Genetics and Developmental Biology, Chinese Academy of Sciences, Beijing, China; ^3^ Department of Botany and Plant Sciences, University of California, Riverside, California, United States

**Keywords:** breast cancer, next generation sequencing, RNA expression, MLPA, 21-gene

## Abstract

RNA in formalin-fixed and paraffin-embedded (FFPE) tissues provides large amount of information indicating disease stages, histological tumor types and grades, as well as clinical outcomes. However, Detection of RNA expression levels in formalin-fixed and paraffin-embedded samples is extremely difficult due to poor RNA quality. Here we developed a high-throughput method, Reverse Transcription-Multiple Ligation-dependent Probe Sequencing (RT-MLPSeq), to determine expression levels of multiple transcripts in FFPE samples. By combining Reverse Transcription-Multiple Ligation-dependent Amplification method and next generation sequencing technology, RT-MLPSeq overcomes the limit of probe length in multiplex ligation-dependent probe amplification assay and thus could detect expression levels of transcripts without quantitative limitations. We proved that different RT-MLPSeq probes targeting on the same transcripts have highly consistent results and the starting RNA/cDNA input could be as little as 1 ng. RT-MLPSeq also presented consistent relative RNA levels of selected 13 genes with reverse transcription quantitative PCR. Finally, we demonstrated the application of the new RT-MLPSeq method by measuring the mRNA expression levels of 21 genes which can be used for accurate calculation of the breast cancer recurrence score – an index that has been widely used for managing breast cancer patients.

## INTRODUCTION

Quantitative changes in RNA expression levels of multiple gene transcripts are commonly used as biomarker signatures in classifying disease status, and in predicting disease progression, prognosis and the effects of interventions [1–3]. There are several approaches to quantify RNA expression. On the genome-wide scale, RNA sequencing and microarray have been widely used to profile the transcriptome and identify the novel biomarkers for the disease phenotypes. However, high cost and requirement for high quality RNA have limited their use in population-scale study and ultimate transfer to routine clinical applications. Based on single-gene assay, reverse transcription-quantitative polymerase chain reaction (RT-qPCR) assay can quantify RNA abundance in both fresh tissue samples and formalin-fixed paraffin-embedded (FFPE) tissue samples with high performance. In the current clinical routine, most solid tissue specimens are preserved using FFPE approach. Thus quantifying the RNA in FFPE tissues could provide valuable information for disease and has great potential for the development of new diagnostic/prognostic assays and therapeutic agents [4–6]. For example, RT-qPCR has been successfully applied to FFPE breast tissues to select 21 prognostic genes from a pool of 250 candidate genes. These 21 genes were used to develop a Recurrence Score (RS) algorithm which can be used to predict patients’ risk of distant recurrence as well as the likelihood of chemotherapy response with estrogen receptor–positive, axillary lymph node-negative breast tumors [2, 3, 7, 8]. Although RT-qPCR has been applied to various clinical diagnostic tests, limited throughput restricts its application to multiple-gene assay. Recent development of microarray technology could also simultaneously quantify hundreds of thousands of diverse mRNA transcripts to paint the full picture of genome-wide expression in FFPE samples [9–11], such as illumina DASL array. However, high cost limits their use in clinical routine.

Reverse-transcriptase multiplex ligation-dependent probe amplification (RT-MLPA) can quantify dozens of transcripts of interest simultaneously [12]. For each target transcript, RT-MLPA uses two oligonucleotides with different lengths which first anneal to adjacent sites on the cDNA of RNA of interest and then are ligated to a joint probe. The joint probe is then amplified by a fluorescence-labelled universal primer pair to obtain amplicons of unique length. Finally the RNA expression is analyzed by capillary electrophoresis system according to their unique length. RT-MLPA technique was derived from MLPA method which was developed for detection of the gain or loss of a single exon or chromosome in human DNA samples [13]. This method has been proved to be sufficiently sensitive, specific and reproductive to quantify 40 or more different target sequences in one tube, regardless of template DNA degradation. The advantages of RT-MLPA give an opportunity to quantify multiple RNA transcripts in FFPE samples. However, due to limitations on the probe length and the fluorescence-dependent analysis, RT-MLPA does not appear to be an efficient method which can analyze hundreds of genes in one reaction.

In the study, we propose a novel high-throughput method, Reverse Transcription-Multiple Ligation-dependent Probe Sequencing (RT-MLPSeq) which combines MLPA method with the next-generation sequencing technology. Length analysis and standard references, which are required in MLPA assay, are no longer needed in the new method. The new method can analyze RNA expression of hundreds of target genes in FFPE samples. Moreover, RT-MLPSeq only needs a small amount of RNA for reaction and has highly concordant results with RT-qPCR.

## RESULTS

### RT-MLPSeq principle

**Figure 1:**
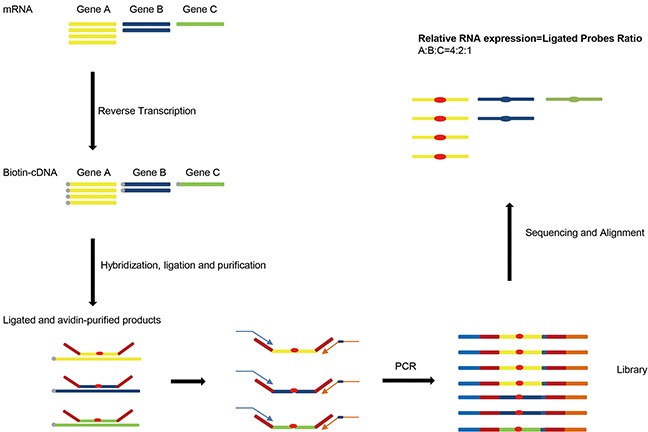
The principle of RT-MLPSeq First RNA from samples were reverse transcribed into cDNA. Probe pairs targeting on different genes were then hybridized to cDNA and ligated by DNA ligase. After PCR with adaptor primers RT-MLPSeq Libraries were prepared and sequenced. Finally relative RNA expression levels of different genes were determined by the ratio of number of corresponding reads.

Due to the probe length limitation, RT-MLPA can only detect up to 45 targets [12] or 90-100 host genes in a single reaction using dual-color assay (dcRT-MLPA) [14]. To improve the throughput and simplify the operating process, we applied Next-Generation Sequencing (NGS) technology to replace the capillary electrophoresis in RT-MLPA method. Compared with standard sequencing system, next-generation sequencing is able to analyze billions of DNA sequences in a single run and could distinguish ligated probes for each target according to the specific sequences of Left Hybridizing Sequence (LHS) and Right Hybridizing Sequence (RHS) in the probes. The overall procedure for RT-MLPSeq were illustrated as Figure 1. RNA from samples were reverse transcribed into cDNA using biotin modified random primer biotin-(dN)_6_ and then hybridized with LPOs and RPOs for target genes. After hybridization and ligation, the ligated probes were purified by streptavidin magnetic beads and used as PCR templates to prepare libraries using P5 and indexed P7 adaptor primers. Libraries from different samples could be pooled into a single sequencing lane and distinguished according to the index contained in the P7 adaptor primers. Sequencing data were aligned to the hybridizing sequences of all gene probes and analyzed to calculate the number of sequence reads that contained both LHS and RHS. Relative RNA expression levels of different genes were determined by the ratio of number of corresponding reads.

### Comparison of RT-MLPSeq to real-time quantification RCR method

**Figure 2:**
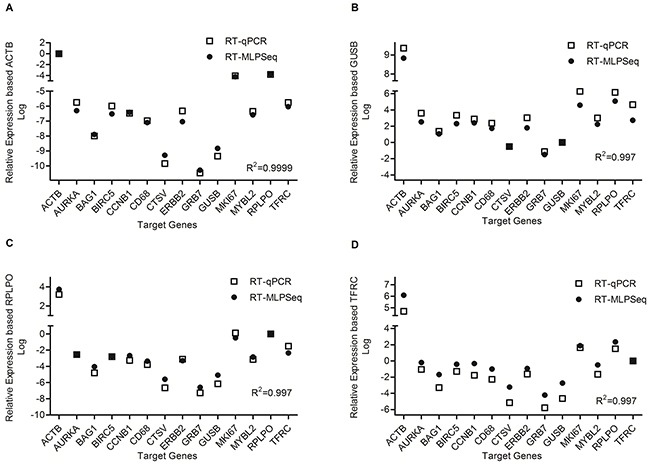
Comparison of RT-MLPSeq to real-time quantification RCR method Four house-keeping gene were selected as reference gene as shown in panel **(A)** (ACTB), panel **(B)** (GUSB), panel **(C)** (RPLPO) as well as panel **(D)** (TFRC). The correlation coefficient were calculated by the default method in GraphPad Prism 5.0 and shown in the lower right of each panel.

Real-time quantification RCR (RT-qPCR) technology has been widely used to quantify RNA expression levels and thus we chose it as standard to compare with RT-MLPSeq method. cDNA reverse-transcribed from RNA of A549 cell lines were hybridized with probe pairs targeting 14 genes, including ACTB, AURKA, BAG1, BIRC5, CCNB1, CD68, CTSV, ERBB2, GRB7, GUSB, MKI67, MYBL2, RPLPO and TFRC. Meanwhile RT-qPCR were performed with SyBr green assay and primer pairs were also selected specifically to the 14 genes. As shown in Figure 2, the relative expression levels of 14 genes from the two methods are in a high agreement, regardless of different house-keeping gene as reference (ACTB, GUSB, RPLPO or TFRC). Among these 10 genes (other than the four house-keeping genes), MKI67 had shown higher RNA expression levels than BIRC5 while GRB7 had the least transcript abundance. This trend was further confirmed in Oncomine™ co-expression database searching for A549 cell line.

### The performance of RT-MLPSeq on different probes targeting the same transcripts

**Figure 3:**
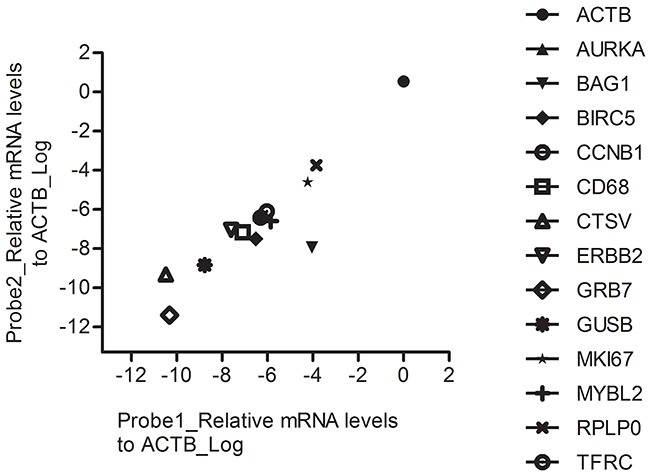
The performance of RT-MLPSeq on two probes targeting the same transcripts RNA expression levels of 14 genes from A549 cell line were determined by RT-MLPSeq using two different probe pairs targeting for the same transcripts. All chosen 14 genes showed similar relative levels between the two probe pairs.

Next we tested the reproducibility of different RT-MLPSeq probes targeting the sample transcripts. To achieve this object, we designed two different probe pairs for each 14 genes, including ACTB, AURKA, BAG1, BIRC5, CCNB1, CD68, CTSV, ERBB2, GRB7, GUSB, MKI67, MYBL2, RPLPO and TFRC. As illustrated in Figure 3, different probe pairs that target the same transcripts showed similar relative levels in A549 cancer cell line.

### Impact of RNA input on performance of RT-MLPSeq

**Figure 4:**
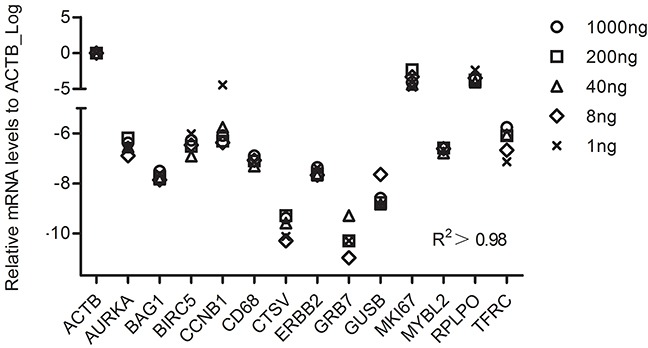
The performance of RT-MLPSeq on different RNA input from same sample Different starting RNA input (1 ng, 8ng, 40ng, 200ng and 1ug RNA from the same A549 sample) were reverse-transcribed and then RNA levels of 13 target genes were determined among these 5 assays. Pearson's correlation analysis were calculated to show the impact of RNA input.

To identity the least RNA needed for the RT-MLPSeq, we performed experiments with starting RNA input from 1 ng to 1 ug. 30 cycles were adopted during the PCR process for the RNA input less than 8 ng and 25 cycles for others. As shown in Figure 4, all the 13 target genes had similar relative expression levels among all the assays with different RNA inputs. Pearson's correlation analysis showed that results from different RNA input were highly relevant (R^2^>0.98).

### Application of RT-MLPSeq to calculate breast cancer recurrence score in FFPE samples

**Figure 5:**
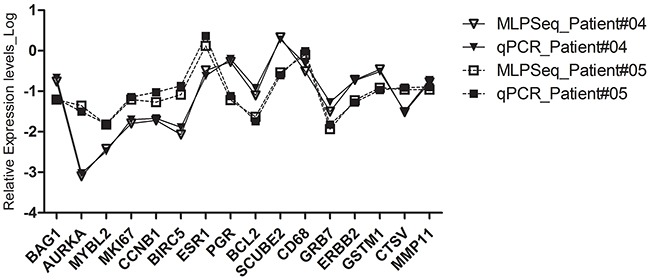
The RNA expression levels of 16 cancer-associated genes in patient #04 and #05 detected by both RT-MLPSeq and RT-qPCT methods The RNA levels of 16 genes, including Ki67, STK15, Survivin, CCNB1, MYBL2, GRB7, HER2, MMP11, CTSL2, ER, PGR, BCL2, SCUBE2, GSTM1, CD68, BAG1 in FFPE sections were normalized to the average Ct value of 5 reference genes (ACTB, GAPDH, RPLPO, GUSB, TFRC).

By using RT-MLPSeq we developed an alternative 21-gene test for lymph node-negative ER-positive breast cancer to predict 10-year recurrence risk and compared with traditional RT-qPCR test. By testing the RNA expression levels in paraffin-embedded breast cancer tissue using RT-qPCR, the 21-gene assay, also called Oncotype^®^ Breast Recurrence Score, is the best-validated prognostic assays for local or distant recurrence and also can predict responses to systemic chemotherapy [2, 3, 15, 16]. All the 21 genes were classified into 6 groups including 16 functional cancer-related genes and 5 reference genes (Supplementary Table 1). To evaluate whether RT-MLPSeq could detect RNA expression levels in the FFPE tissues and further get the exact recurrence score as RT-qPCR methods, we designed two pairs of RT-MLPSeq probes for each gene (Supplementary Table 2) and detected these mRNA levels of the 21 genes in FFPE sections from 11 breast cancer patients in our hospital with both RT-MLPSeq and RT-qPCR methods. All these patients were postmenopausal women with ER-positive breast cancer and their information were shown in Table 1. Most RNA extracted from FFPE breast cancer tissues appeared to be highly fragmented below 100bp (shown in Supplementary Figure 1). Probe pairs targeting on 21 genes were then hybridized to cDNA and ligated by Taq DNA ligase. After PCR with P5/P7 adaptor primers RT-MLPSeq Libraries from FFPE RNA were successfully prepared (Supplementary Figure 2) and sequenced. Meanwhile we applied RT-qPCR methods to measure RNA expression as previous reported [14] and compared with results derived from RT-MLPSeq for the same sample. The mRNA expression levels of 16 cancer-associated genes (Ki67, STK15, Survivin, CCNB1, MYBL2, GRB7, HER2, MMP11, CTSL2, ER, PGR, BCL2, SCUBE2, GSTM1, CD68, BAG1) detected by both methods were normalized to 5 reference genes (ACTB, GAPDH, RPLPO, GUSB, TFRC). As shown in Figure 5, similar expression levels were detected by RT-MLPSeq and RT-qPCR methods for the same genes in the same sample. Furthermore, RNA expression levels detected by RT-MLPSeq were consistent with the immunohistochemistry (IHC) staining results. For instance, The PR protein expressed at least 90% of the breast cancer cells of patient #4 while only 10% in the patient #5 (Table 1). This difference was seen at the mRNA levels of PR using RT-MLPSeq and RT-qPCR methods which showed that RNA levels of PR in patient #4 were higher than that of patient #5 (Figure 5). Another example is MKI67 gene, which had higher expression at protein level (40% vs 10%, Table 1) and RNA levels (Figure 5) in patient #5 than that of patient #4. All these results indicated that RT-MLPSeq can be used as an accurate method to detect RNA expression levels in FFPE samples.

**Table 1:** Patients information

Patient	Gender	Age	Tumor type	Clinical stage	Tumor size	IHC
1	Female	58	Invasive breast carcinoma	pT2N0	3.0×2.8×2.0cm	CK5&6(−), E-cadherin(1+), EGFR(−), ER(+,95%), HER2(2+), Ki-67(+,30%), P53(−), PR(+, 60%), TOP2A(2+)
2	Female	53	Invasive breast carcinoma	pT1cN0	1.2×1.2×1.0cm	CK5&6(−), E-cadherin(2+), EGFR(−), ER(+, >90%), HER2(1+), Ki-67(+, 25%), PR(+, 10%)
3	Female	54	Invasive breast carcinoma	pT1cN0	1.5×1.2×1.2cm	CK5&6(−), E-cadherin(2+), EGFR(−), ER(+80%), HER2(2+), Ki-67(+20-30%), P53(−), PR(+10%), TOP2A(1+)
4	Female	47	Invasive lobular carcinoma	pT1cN0	1.4×1.2×1.2cm	CK5&6(−), E-cadherin(−), EGFR(−), ER(+, >95%), HER2(1+), Ki-67(+, 10%), P53(−), PR(+, >95%), TOP2A(1+)
5	Female	64	Invasive breast carcinoma	pT1N0	1.8×1.5×1.3cm	CK5&6(−), E-cadherin(1+), EGFR(−), ER(+80%), HER2(1+), Ki-67(+40%), P53(−), PR(+, <10%), TOP2A(2+)
6	Female	38	Invasive breast carcinoma	pT1cN0	1.8×1.5×1.3cm	CK5&6(−), E-cadherin(3+), EGFR(−), ER(+, 80%), HER2(1+), Ki-67(20%+), P53(−), PR(+, 80%), TOP2A(10%+)
7	Female	38	Invasive breast cancer with focal DCIS	pT1N0	2.2×1.8×1.3cm	CK5&6(−), E-cadherin(3+), EGFR(−), ER(+, >90%), HER2(1+), Ki-67(+5%), P53(−), PR(+, 25%)
8	Female	43	Invasive breast cancer with focal DCIS	pT1bN0	1.0×0.5×0.5cm	CK5&6(−), E-cadherin(2+), EGFR(−), ER(+, >90%), HER2(1+), Ki-67(+, 30%), P53(2+), PR(+, 70%), TOP2A(1+)
9	Female	63	Invasive ductal carcinoma	pT1N0	1.8×1.3×1.1cm	CK5/6(−), E-cad(2+), EGFR(1+), ER(+90%), HER2(2+), Ki-67(+, 30%), P53(−), PR(+1-10%), Top2A(1+)
10	Female	57	Invasive breast carcinoma	pT1N0	1.3×1.0×1.0cm	CK5&6(−), E-cadherin(2+), EGFR(−), ER(80%), HER2(1+), Ki-67(30%), P53(+), PR(80%), TOP2A(1+)
11	Female	53	Invasive breast carcinoma	pT1cN0	1.4×1.4×1.2cm	CK5&6(−), E-cadherin(3+), EGFR(−), ER(+, >95%), HER2(−), Ki-67(+, 30%), P53(−), PR(+, 80%), TOP2A(2+)

**Figure 6:**
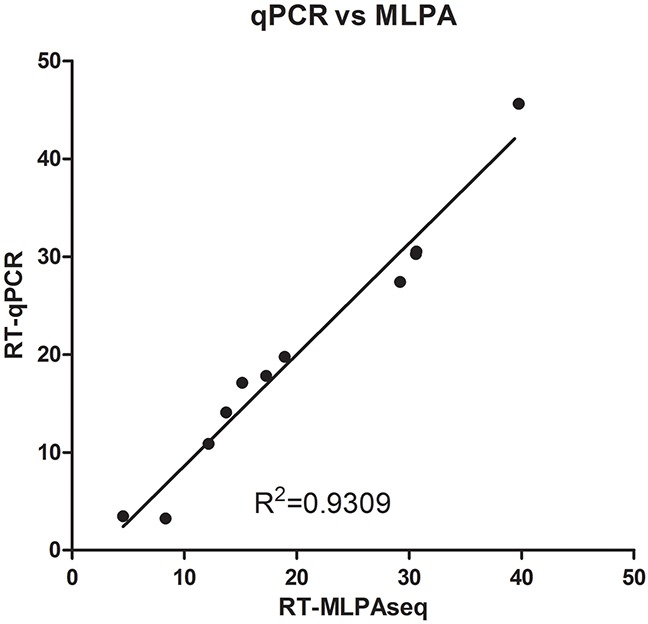
Recurrence scores of 11 breast cancer patients calculated by RT-qPCR and RT-MLPSeq methods RNA from FFPE sections of 11 breast cancer patients were extracted and reverse-transcribed into cDNA. Then cDNA from the same patients were separated to determine the relative expression levels of 21 gene by RT-qPCR and MLPSeq. Recurrence scores were finally calculated according to the algorithm presented by Paik et al.

To calculate the recurrence score, we converted the relative transcripts expression fold from both RT-MLPSeq method and RT-qPCR method to ΔCt value (−log_2_) and finally obtained the RS value based on the algorithm presented by Paik, Shak, Tang, et al. in which ΔCt values from real time PCR system were used as input data [3]. As shown in Figure 6, recurrence scores from RT-MLPSeq was highly consistent with RT-qPCR results (R^2^=0.9309).

## DISCUSSION

Currently the most commonly used RNA quantification methods in FFPE samples were RT-qPCR [4, 5, 14]. To our best knowledge, the length of RNA from FFPE samples can be as small as 80 bps [6], which could be reverse-transcribed to cDNA larger than 60 bps. MLPA were designed to detect copy number and methylation changes of DNA, as well as relative expression levels of RNA. The detection procedure including hybridization probes to DNA templates, ligation of matched probe pairs and amplification with universal PCR primers. For RT-MLPA analysis, the length of cDNA template may be small because probe pairs need only about 60 bps long template to be hybridized during hybridization and ligation steps. Here we reported that RT-MLPSeq, a method derived from RT-MLPA, could also detect relative RNA levels in FFPE samples. We compared RT-MLPSeq with RT-qPCR and proved that there was high level of consistence between these two methods.

The other feature of RT-MLPSeq was high-throughput for target RNA. Unlike traditional RT-MLPA [12] or dual-color RT-MLPA [14] which distinguish different targets with unique length of ligated probe pairs, NGS can analyze the specific nucleotide sequences in LHS and RHS and thus have no limit on the number of probes in one reaction.

There were other next generation sequencing methods to detect RNA profiling, such as RNAseq, DeepSAGE [17], RASL [18] and RASL-seq [19]. RNAseq and DeepSAGE detect the whole transcriptome and thus are expensive compared with the target sequencing. Similar with RT-MLPSeq, RASL-seq is another method that could detect the expressional levels of targeted transcripts based on ligation reaction. However, RASL-seq uses poly-T coated magnetic beads to purify mRNA and ligated probes for next amplification and thus need RNA with high integrity, which means it could not be used on FFPE samples. Furthermore, other than RASL-seq using customized sequencing primers for sequencing, RT-MLPSeq uses the Illumina-compatible sequencing primers and thus had more flexible pooling strategy, especially when RT-MLPSeq needs extremely little data (below 1M reads).

In summary, we developed a high-throughput method to detect RNA expression levels in FFPE samples with the starting RNA input as little as 1 ng. Considering its low cost, highly flexible throughput and ability to handle FFPE tissue, it can be used in both investigational researches and clinical labs.

## METHODS

### Probe design

**Figure 7:**
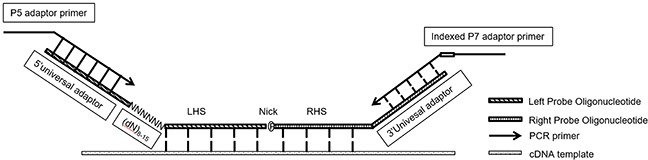
RT-MLPSeq probes design P5 adaptor primer contained identical sequences of illumina P5 Sequence and 5′ universal adaptors while indexed P7 adaptor primer had reverse-complemental sequences of illumina P7 and 3′ universal adaptor as well as 8Nt index.

Normally we design MLPA probes according to “Designing synthetic MLPA probes” from MRC-Holland except PCR primer sequences. MLPA probe pair for each target including Left Probe Oligonucleotide (LPO) and Right Probe Oligonucleotide (RPO). To prepare sequencing libraries for NGS, we re-designed the MLPA probes as shown in Figure 7. Unlike standard MLPA probes, we removed the optional stuffer on both LPO and RPO and replaced forward/reverse primer binding sites as 5′ universal adaptor sequences (5′TACACTCTTT CCCTACACGA CGCTCTTCCG ATCT3′) and 3′ universal adaptor sequences (5′AGATCGGAAG AGCACACGTC TGAACTCCAG TCAC3′), which function as sequencing primers in the NGS step. We also added (dN)_8-15_ random sequences at the 5′ end of LHS as Unique Identifier (UID) to eliminate PCR bias in the further analysis.

LPO includes an adaptor sequence for Illumina at 5′ end and Left Hybridizing Sequence (LHS) at 3′ end. RPO contains Right Hybridizing Sequence (RHS) at 5′ end and an adaptor sequence at 3′ end. LHS and RHS should be directly adjacent and exactly reverse-complimentary to cDNA from target RNA. The design of LHS and RHS follow the below rules:

1) 26-40 nts length, Tm >70°C;

2) Span exon;

3) GC content is between 40%-60%;

4) No or limited homology with other human sequences;

5) The last nucleotide of the LPO has a mismatch with the related sequence;

6) No SNP or the SNP effect is minimized by placing SNP at least 8 nts from the ligation site.

### RNA extraction, cDNA synthesis and real-time qPCR

Total RNA were extracted from FFPE cancer tissues or A549 cell line using Trizol reagent (Invitrogen) following the supplier's protocol and first-strand cDNA were synthesized using PrimeScript II 1^st^ Strand cDNA Synthesis kit (Takara) with biotin-modified (dN)_6_ primer. Relative quantitative Real-Time PCR were carried out on ABI 7500 Real-time PCR system using All-in-one™ Real-Time PCR Master Mix (Genecopoeia) with 2^−△△CT^ method with selected reference genes. The sequences of primers were shown as previously report [20].

### Library preparation and sequencing on Illumina sequencing platform

MLPA reactions were performed in a thermocycler with a heated lid. First, biotin-modified cDNA were mixed with probes mix and 1 unit Taq ligase in 20 μl 1X Taq ligase buffer and then were put in thermocycler following program 95°C for 5 min, 60°C for 90 min and saved at 4°C before use. Second, the ligation products were then purified by streptavidin-magnetic beads (NEB) and used as template for next PCR steps. Third, purified ligation products were amplified with primer pairs containing P5/P7 sequences and unique index to distinguish different samples in a single sequencing run. Finally PCR products were purified using Beckmann AMPure XP beads to remove primer dimers and libraries were ready for sequencing. Prepared libraries were pooled and sequenced on Illumina HiSeq2500 with SE50 strategy or NextSeq 500 with PE75 for 1M reads.

### Patient information and recurrence score calculation

All ER^+^ FFPE sections were collected from volunteers who had undergone surgery for breast cancer at the Cancer Hospital, Chinese Academy of Medical Sciences between October 2012 and March 2016. These patients have been well informed by telephone follow-up. The clinical and laboratory data of these patients were gathered from medical records, including age, gender, tumor type, clinical stage, tumor size as well as IHC status. Collection of these sections and data were approved by the ethics committees of Cancer Hospital, Chinese Academy of Medical Sciences and Peking Union Medical College.

RNA was extracted from FFPE breast cancer tissues (QIAGEN RNeasy FFPE Kit) and was reverse-transcribed with random Biotin-(dN)_6_ primers. For RT-qPCR methods, the cDNA from FFPE samples and 21-gene primers as previously reported [14] were added to All-in-one™ Real-Time PCR Master Mix (Genecopoeia) and then analyzed on ABI 7500 Real-time PCR system. For RT-MLPSeq method, probe pairs targeting on the 21 genes were then hybridized to the same cDNA used in RT-qPCR method and ligated by Taq DNA ligase. After PCR with P5/P7 adaptor primers RT-MLPSeq Libraries from FFPE RNA were sequenced on NextSeq 500 with PE75 strategy. After filtered, reads mapped to the target sequences of 21 genes were counted and regarded as expression levels. After converting the relative expression fold to traditional ΔCt value used in real time PCR system(−log2), recurrence score were calculated according to the algorithm presented by Paik S, Shak S, Tang G, et al [2].

### Data analysis

After base calling and data filtration, sequencing data for each sample was separated from raw data according to index sequences using illumina cassava pipeline. The single-end reads were then aligned to the hybridizing sequences and counted as the hit reads numbers with homebrew software. Relative expression for specific genes were then calculated based on the hit reads number ratio between target genes and the selected reference genes. All graph and relative data analysis were performed in GraphPad Prism 5.0 software using default settings.

## SUPPLEMENTARY MATERIALS FIGURES AND TABLES




