# Supplementary material for: A high-throughput method to detect RNA profiling by integration of RT-MLPA with next generation sequencing technology
Source: Oncotarget. 2017 May 2;8(28):46071–80. doi: 10.18632/oncotarget.17551 (PMC5542250; doi:10.18632/oncotarget.17551)
Supplement: Supplementary file 1 [file oncotarget-08-46071-s001.pdf]

# A high-throughput method to detect RNA profiling by integration of RT-MLPA with next generation sequencing technology

## SUPPLEMENTARY MATERIALS

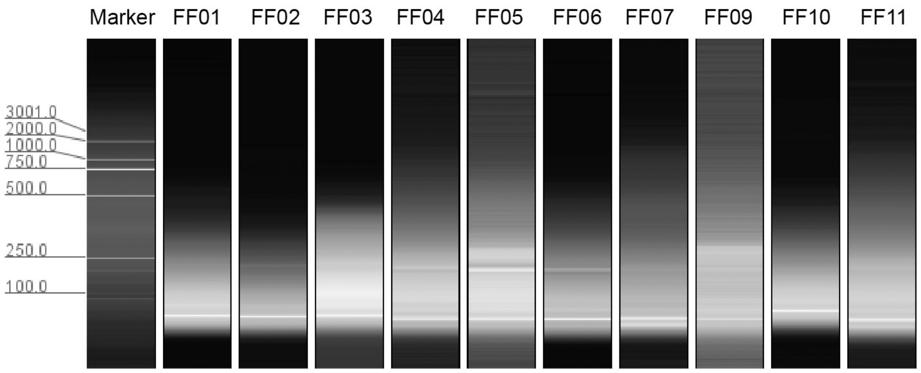


**Supplementary Figure 1: FFPE RNA length distribution.**

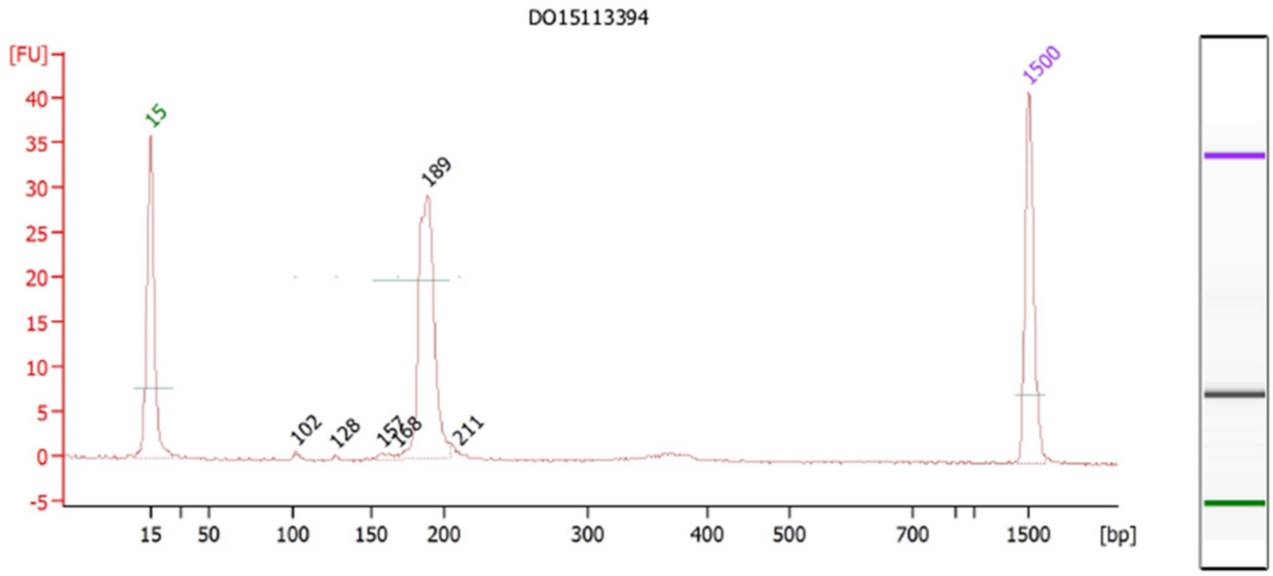


**Supplementary Figure 2: Length analysis of RT-MLPSeq libraries from FFPE RNAs.**

**Supplementary Table 1: The gene symbols involved 21-gene test**

| | |
|---|---|
| Proliferation group | Ki67, STK15, Survivin, CCNB1, MYBL2 |
| HER2 group | GRB7, HER2 |
| Invasion group | MMP11, CTSL2 |
| Estrogen group | ER, PGR, BCL2, SCUBE2 |
| Reference group | ACTB, GAPDH, RPLPO, GUSB, TFRC |
| Others | GSTM1, CD68, BAG1 |

**Supplementary Table 2. The sequences of RT-MLPSeq probes for the 21gene assay**

**See Supplementary File 1**
