# Supplementary material for: A high-throughput method to detect RNA profiling by integration of RT-MLPA with next generation sequencing technology
Source: Oncotarget. 2017 May 2;8(28):46071–80. doi: 10.18632/oncotarget.17551 (PMC5542250; doi:10.18632/oncotarget.17551)
Supplement: Supplementary file 2 [file oncotarget-08-46071-s002.docx]

Table S2. The sequences of RT-MLPSeq probes for the 21gene assay.

| Symbol | LPO/RPO |
| --- | --- |
| ACTBL2 | TACACTCTTTCCCTACACGACGCTCTTCCGATCTTGGCACCCAGCACAATGAAGATCAAGA |
| ACTBL3 | TACACTCTTTCCCTACACGACGCTCTTCCGATCTAGGGCGTGATGGTGGGCATGGGTCA |
| ACTBR2 | TCATTGCTCCTCCTGAGCGCAAGTACTCAGATCGGAAGAGCACACGTCTGAACTCCAGTCAC |
| ACTBR3 | GAAGGATTCCTATGTGGGCGACGAGGAGATCGGAAGAGCACACGTCTGAACTCCAGTCAC |
| AURKAL1 | TACACTCTTTCCCTACACGACGCTCTTCCGATCTTCAGCTCAGAAGAGAAGTAGAAATACAGTCCCA |
| AURKAL2 | TACACTCTTTCCCTACACGACGCTCTTCCGATCTAAGAGGCAGTGGGCTTTGGAAGACTTTGAA |
| AURKAR1 | CCTTCGGCATCCTAATATTCTTAGACTGTATGGAGATCGGAAGAGCACACGTCTGAACTCCAGTCAC |
| AURKAR2 | ATTGGTCGCCCTCTGGGTAAAGGAAAGTAGATCGGAAGAGCACACGTCTGAACTCCAGTCAC |
| BAG1L1 | TACACTCTTTCCCTACACGACGCTCTTCCGATCTTGAGGCACAAGAATCATTTGAACCTGGGAG |
| BAG1L2 | TACACTCTTTCCCTACACGACGCTCTTCCGATCTGCAATGAGAAGCACGACCTTCATGTTACCT |
| BAG1R1 | GTAGAGGTTGCTGTGAGCCGAGATTACGCCAGATCGGAAGAGCACACGTCTGAACTCCAGTCAC |
| BAG1R2 | CCCAGCAGGGCAGCAGTGAACCAGTAGATCGGAAGAGCACACGTCTGAACTCCAGTCAC |
| BCL2L1 | TACACTCTTTCCCTACACGACGCTCTTCCGATCTCTTGACAGAGGATCATGCTGTACTTAAAAAATACAAC |
| BCL2L2 | TACACTCTTTCCCTACACGACGCTCTTCCGATCTGGATCCAGGATAACGGAGGCTGGGAT |
| BCL2R1 | ATCACAGAGGAAGTAGACTGATATTAACAATACTTACAGATCGGAAGAGCACACGTCTGAACTCCAGTCAC |
| BCL2R2 | GCCTTTGTGGAACTGTACGGCCCCAAGATCGGAAGAGCACACGTCTGAACTCCAGTCAC |
| BIRC5L1 | TACACTCTTTCCCTACACGACGCTCTTCCGATCTGAGGCTGGCTTCATCCACTGCCCCACTGAGA |
| BIRC5L2 | TACACTCTTTCCCTACACGACGCTCTTCCGATCTTAAGTCATTGGGGAAACGGGGTGAACTTCAG |
| BIRC5R1 | ACGAGCCAGACTTGGCCCAGTGTTTCTTCTAGATCGGAAGAGCACACGTCTGAACTCCAGTCAC |
| BIRC5R2 | GTGGATGAGGAGACAGAATAGAGTGATAGGAAGCGAGATCGGAAGAGCACACGTCTGAACTCCAGTCAC |
| CCNB1L1 | TACACTCTTTCCCTACACGACGCTCTTCCGATCTTGACTGTCTCCATTATTGATCGGTTCATGC |
| CCNB1L2 | TACACTCTTTCCCTACACGACGCTCTTCCGATCTGCAGCACCTGGCTAAGAATGTAGTCATGGTAA |
| CCNB1R1 | AGAATAATTGTGTGCCCAAGAAGATGCTGCAGATCGGAAGAGCACACGTCTGAACTCCAGTCAC |
| CCNB1R2 | ATCAAGGACTTACAAAGCACATGACTGTCAAGAGATCGGAAGAGCACACGTCTGAACTCCAGTCAC |
| CD68L1 | TACACTCTTTCCCTACACGACGCTCTTCCGATCTCACCTGCTTCTCTCATTCCCCTATGGACA |
| CD68L2 | TACACTCTTTCCCTACACGACGCTCTTCCGATCTTCATTCTTTCACCAGCTGTCCACCTCGACC |
| CD68R1 | CCTCAGCTTTGGATTCATGCAGGACCTCAGATCGGAAGAGCACACGTCTGAACTCCAGTCAC |
| CD68R2 | TGCTCTCCCTGAGGCTCCAGGCTGCTCAAGATCGGAAGAGCACACGTCTGAACTCCAGTCAC |
| CTSVL1 | TACACTCTTTCCCTACACGACGCTCTTCCGATCTGGAGGAGTTTGCTGATGTGGGAGTGTAGTTATG |
| CTSVL2 | TACACTCTTTCCCTACACGACGCTCTTCCGATCTCACAGAAGATTATATGGCGCGAATGAAGAAG |
| CTSVR1 | ACACTTGGGCATCATACTAGAGGCTATGGACTTAGATCGGAAGAGCACACGTCTGAACTCCAGTCAC |
| CTSVR2 | GATGGAGGAGAGCAGTGTGGGAAAAGAATAAGATCGGAAGAGCACACGTCTGAACTCCAGTCAC |
| ERBB2L1 | TACACTCTTTCCCTACACGACGCTCTTCCGATCTGAAGTGCAGCAAGCCCTGTGCCCGAGTGT |
| ERBB2L2 | TACACTCTTTCCCTACACGACGCTCTTCCGATCTAGCTCCAAGTGTTTGAGACTCTGGAAGAGA |
| ERBB2R1 | GCTATGGTCTGGGCATGGAGCACTTGCGAGAAGATCGGAAGAGCACACGTCTGAACTCCAGTCAC |
| ERBB2R2 | TCACAGGTTACCTATACATCTCAGCATGGCAGATCGGAAGAGCACACGTCTGAACTCCAGTCAC |
| ESR1L1 | TACACTCTTTCCCTACACGACGCTCTTCCGATCTACATGAGTAACAAAGGCATGGAGCATCTGTA |
| ESR1L2 | TACACTCTTTCCCTACACGACGCTCTTCCGATCTGACATGCTGCTGGCTACATCATCTCGGTTC |
| ESR1R1 | CAGCATGAAGTGCAAGAACGTGGTGCCCCTAGATCGGAAGAGCACACGTCTGAACTCCAGTCAC |
| ESR1R2 | CGCATGATGAATCTGCAGGGAGAGGAGTTTAGATCGGAAGAGCACACGTCTGAACTCCAGTCAC |
| GAPDHL1 | TACACTCTTTCCCTACACGACGCTCTTCCGATCTCTCAACGACCACTTTGTCAAGCTCATTTCCT |
| GAPDHL2 | TACACTCTTTCCCTACACGACGCTCTTCCGATCTCGATTTCTCCTCCGGGTGATGCTTTTCCTA |
| GAPDHR1 | GGTATGACAACGAATTTGGCTACAGCAACAGGAGATCGGAAGAGCACACGTCTGAACTCCAGTCAC |
| GAPDHR2 | GATTATTCTCTGATTTGGTCGTATTGGGCGAGATCGGAAGAGCACACGTCTGAACTCCAGTCAC |
| GRB7L1 | TACACTCTTTCCCTACACGACGCTCTTCCGATCTTCTACGGGATGCCCACTGACTTCGGTTTC |
| GRB7L2 | TACACTCTTTCCCTACACGACGCTCTTCCGATCTGGGCTTTGTCCTCTCTTTGTGCCACCTG |
| GRB7R1 | TGTGTCAAGCCCAACAAGCTTCGAAATGGCAGATCGGAAGAGCACACGTCTGAACTCCAGTCAC |
| GRB7R2 | CAGAAAGTGAAGCATTATCTCATCCTGCCGAGATCGGAAGAGCACACGTCTGAACTCCAGTCAC |
| GSTM1L2 | TACACTCTTTCCCTACACGACGCTCTTCCGATCTAAGCACAACCTGTGTGGGGAGACAGAAG |
| GSTM1L3 | TACACTCTTTCCCTACACGACGCTCTTCCGATCTTTCCTCCCAAGACCTGTGTTCTCAAAGATG |
| GSTM1R2 | AGGAGAAGATTCGTGTGGACATTTTGGAGAAGATCGGAAGAGCACACGTCTGAACTCCAGTCAC |
| GSTM1R3 | GCTGTCTGGGGCAACAAGTAGGGCCTTGAAGATCGGAAGAGCACACGTCTGAACTCCAGTCAC |
| GUSBL1 | TACACTCTTTCCCTACACGACGCTCTTCCGATCTTCCCACCTAGAATCTGCTGGCTACTACTTGAAG |
| GUSBL2 | TACACTCTTTCCCTACACGACGCTCTTCCGATCTTATGGAGCAGAAACGATTGCAGGGTTTCAC |
| GUSBR1 | ATGGTGATCGCTCACACCAAATCCTTGGACAGATCGGAAGAGCACACGTCTGAACTCCAGTCAC |
| GUSBR2 | CAGGATCCACCTCTGATGTTCACTGAAGAGTAAGATCGGAAGAGCACACGTCTGAACTCCAGTCAC |
| MKI67L1 | TACACTCTTTCCCTACACGACGCTCTTCCGATCTTGCTCCCCACCTCAGAGAGTTTTGGAGGAA |
| MKI67L2 | TACACTCTTTCCCTACACGACGCTCTTCCGATCTCACTCCACCTGTCCTGAAGAAAATCATCAA |
| MKI67R1 | ATGTGTTCTTCAGTGCACAGAATGCAGCAAAACAGATCGGAAGAGCACACGTCTGAACTCCAGTCAC |
| MKI67R2 | GGAACAGCCTCAACCATCAGGAAAACAAGAAGATCGGAAGAGCACACGTCTGAACTCCAGTCAC |
| MMP11L1 | TACACTCTTTCCCTACACGACGCTCTTCCGATCTTGCCCTCTGAGATCGACGCTGCCTTCCA |
| MMP11L2 | TACACTCTTTCCCTACACGACGCTCTTCCGATCTTGGGAGAAGACGGACCTCACCTACAGGA |
| MMP11R1 | GGATGCTGATGGCTATGCCTACTTCCTGCGAGATCGGAAGAGCACACGTCTGAACTCCAGTCAC |
| MMP11R2 | TCCTTCGGTTCCCATGGCAGTTGGTGCAAGATCGGAAGAGCACACGTCTGAACTCCAGTCAC |
| MYBL2L1 | TACACTCTTTCCCTACACGACGCTCTTCCGATCTGCCCTGGACTTGATCGAGTCGGACCCTGAT |
| MYBL2L2 | TACACTCTTTCCCTACACGACGCTCTTCCGATCTCAACCGCTGGGCCGAGATCGCCAAGATGT |
| MYBL2R1 | GCTTGGTGTGACCTGAGTAAATTTGACCTCCCTGAGATCGGAAGAGCACACGTCTGAACTCCAGTCAC |
| MYBL2R2 | TGCCAGGGAGGACAGACAATGCTGTGAAGAAGATCGGAAGAGCACACGTCTGAACTCCAGTCAC |
| PGRL1 | TACACTCTTTCCCTACACGACGCTCTTCCGATCTCAACTTACAAAACTTCTTGATAACTTGCATGATCTTG |
| PGRL2 | TACACTCTTTCCCTACACGACGCTCTTCCGATCTACAGATAGTCATGTGTTTGATGATGGGCAC |
| PGRR1 | TCAAACAACTTCATCTGTACTGCTTGAATACATTTATCCAGATCGGAAGAGCACACGTCTGAACTCCAGTCAC |
| PGRR2 | TGTGGAGATAACTGACATAGGACTGTGCCCAGATCGGAAGAGCACACGTCTGAACTCCAGTCAC |
| RPLP0L1 | TACACTCTTTCCCTACACGACGCTCTTCCGATCTTACACCTTCCCACTTGCTGAAAAGGTCAAG |
| RPLP0L2 | TACACTCTTTCCCTACACGACGCTCTTCCGATCTTGCTGATGGGCAAGAACACCATGATGCG |
| RPLP0R1 | GCCTTCTTGGCTGATCCATCTGCCTTTGTGAGATCGGAAGAGCACACGTCTGAACTCCAGTCAC |
| RPLP0R2 | CAAGGCCATCCGAGGGCACCTGGAAAACAAGATCGGAAGAGCACACGTCTGAACTCCAGTCAC |
| SCUBE2L1 | TACACTCTTTCCCTACACGACGCTCTTCCGATCTCAGATGAGAAGTCTTGCCAAGATGTGGATGAG |
| SCUBE2L2 | TACACTCTTTCCCTACACGACGCTCTTCCGATCTAGCTGCCATCCACAGTACAAGATGCACACA |
| SCUBE2R1 | TGCTCTTTGGATAGGACCTGTGACCACAGCAGATCGGAAGAGCACACGTCTGAACTCCAGTCAC |
| SCUBE2R2 | GATGGGAGGAGCTGCCTTGAGCGAGAAGATCGGAAGAGCACACGTCTGAACTCCAGTCAC |
| TFRCL1 | TACACTCTTTCCCTACACGACGCTCTTCCGATCTGTGTGGCAGTTCAGAATGATGGATCAAGCT |
| TFRCL2 | TACACTCTTTCCCTACACGACGCTCTTCCGATCTCAGCCAACTGCTTTCATTTGTGAGGGAT |
| TFRCR1 | AGATCAGCATTCTCTAACTTGTTTGGTGGAGAACAGATCGGAAGAGCACACGTCTGAACTCCAGTCAC |
| TFRCR2 | CTGAACCAATACAGAGCAGACATAAAGAAATGAGATCGGAAGAGCACACGTCTGAACTCCAGTCAC |
